# Limitations of Sulforhodamine 101 for Brain Imaging

**DOI:** 10.3389/fncel.2017.00044

**Published:** 2017-02-28

**Authors:** Swen Hülsmann, Liya Hagos, Heike Heuer, Christian Schnell

**Affiliations:** ^1^Clinic for Anesthesiology, University Hospital GöttingenGöttingen, Germany; ^2^DFG Research Center for Nanoscale Microscopy and Molecular Physiology of the Brain (CNMPB)Göttingen, Germany; ^3^Leibniz-Institut für Umweltmedizinische Forschung GmbHDüsseldorf, Germany

**Keywords:** astrocytes, neurosteroids, thyroxine, sulforhodamine 101, imaging

## Abstract

Since 2004, the red fluorescent dye Sulforhodamine 101 (SR101) has been boosting the functional analysis of astrocytes in a functional environment in an unprecedented way. However, two major limitations have been challenging the usefulness of this tool for cellular imaging: (i) SR101 is not as specific for astrocytes as previously reported; and (ii) discoveries of severe excitatory side effects of SR101 are bearing the risk of unwanted alteration of the system of interest. In this article, we summarize the current knowledge about SR101-labeling protocols and discuss the problems that arise from varying of the staining protocols. Furthermore, we provide a testable hypothesis for the observed hyper-excitability that can be observed when using SR101.

The red fluorescent dye, Sulforhodamine 101 (SR101), is a rather old tool for life scientists. First used for flow cytometry already in 1978 (Stöhr et al., 1978), it later appeared to be helpful for labeling of active synapses (Lichtman et al., 1985), as well as neurons and astrocytes in intact preparations (Cina and Hochman, 2000). The description of “Sulforhodamine 101 as a specific marker of astroglia in the neocortex *in vivo*” in 2004 (Nimmerjahn et al., 2004) boosted the research on astroglial cells. However, many aspects, including the ultimate mechanism of SR101 uptake into astrocytes remained unknown and, furthermore, the method has been challenged by a lack of cell type specificity and reports of excitatory side effects. Since the method is used by a still growing number of research groups, we summarize the available information of SR101-staining and its limitation with respect to cell specificity from the current literature and discuss the excitatory side effect in the context of recent publications and our own experimental data, which point towards a role of neurosteroids in the generation of SR101-induced hyper-excitability.

## Fluorescent Labeling Protocol and Type of Labeling

**Figure 1:**
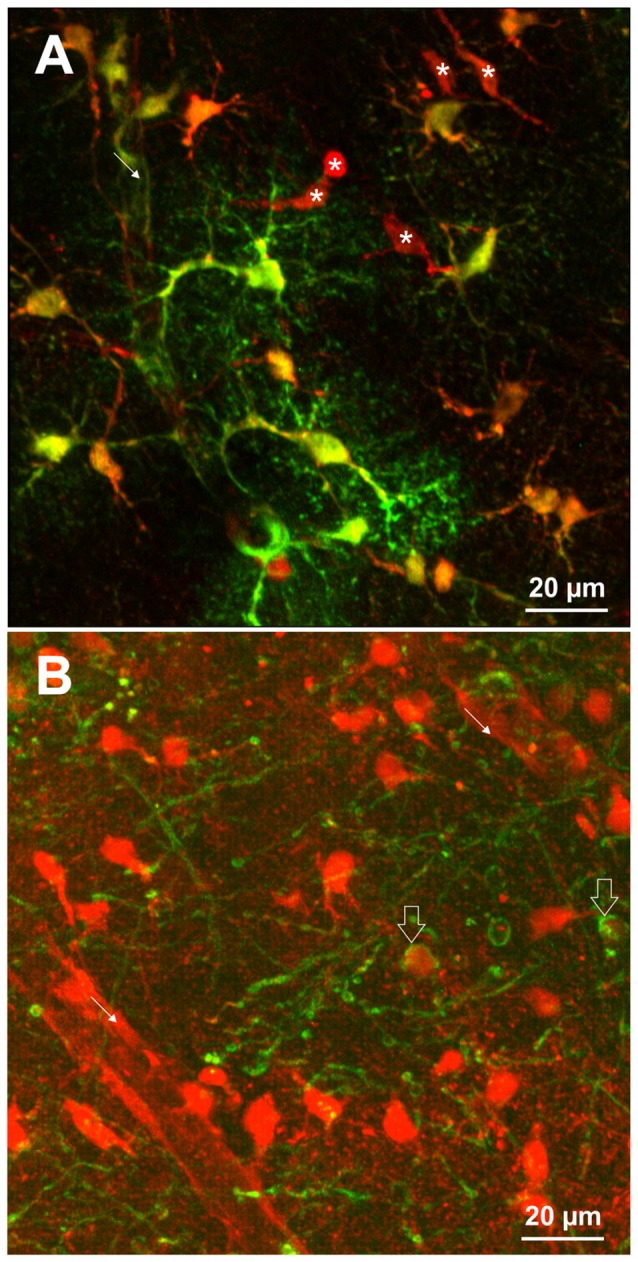
**(A)** SR101-labeling of astrocytes identified by transgenic expression of EGFP (green) using TgN(hGFAP-EGFP)GFEC-Fki; (Nolte et al., 2001). Incubation of the brain slice with 1 μM SR101 for 20 min at 34°C followed 10 min of de-staining in artificial cerebrospinal fluid (aCSF) reveals that also some cells are nicely labeled by SR101 but do not express the astrocyte marker (asterisk). Note that the SR101 fluorescence is reaching the end feet of the astrocytes (fine arrow), but is weak in the distal processes of the astrocytes. **(B)** SR101-staining of oligodendrocytes that were identified by transgenic expression of EGFP using TgN(PLP-GFP) mice (Spassky et al., 2001). Slices were incubated in 1 μM SR101 for 140 min at 34°C followed 10 min of de-staining in aCSF. This method reveals that also cells from the oligodendrocyte linage (open arrows) are labeled by SR101. The intensity of SR101 is weaker as compared to neighboring GFP-negative cells. Image acquisition using 2-Photon excitation microscopy was described previously for astrocytes (Schnell et al., 2012) and oligodendrocytes (Hagos and Hülsmann, 2016). Pictures are surface mode projections of 100 μm image stacks generated by Imaris software (Bitplane).

Different staining protocols have been used to label astrocytes. For *in vivo* imaging, SR101 was applied topically at concentrations of 250 nM to 300 μM or by bolus injection (Nimmerjahn et al., 2004; Nimmerjahn and Helmchen, 2012). Additionally, SR101 injection over the tail vein (10 mg/ml) has been reported to be successful (Appaix et al., 2012). Acute brain slices are usually incubated in carbonated extracellular solution containing 0.5–1 μM SR101 for 20–30 min and 34–37°C. Following this, excess dye is removed over a period of 10–30 min using different protocols that were described earlier (Kafitz et al., 2008; Meier et al., 2008; Kantor et al., 2012; Schnell et al., 2012, 2015; Augustin et al., 2016; Hagos and Hülsmann, 2016). These protocols lead to labeling of cell somata and proximal processes of astrocytes. The fine distal processes of astrocytes as revealed e.g., by transgenic expression of fluorescent protein, are often difficult to identify by SR101 (see Figure 1A). If the staining is weaker (e.g., in the brainstem), proximal processes appear unlabeled.

## The Problem of Cell Type Specificity

SR101 became extremely important for the study of astrocytes after the publication by Nimmerjahn et al. (2004). For the relatively novel field of glia-physiology that was always depending on genetically engineered mice to label the cell type of interest for physiological studies with a fluorescent protein (Nolte et al., 2001) or, before these animals became available, by *post hoc* immunohistochemical counterstaining of dye-filled cells with antibodies against astroglial marker proteins, e.g., GFAP (Konietzko and Müller, 1994), SR101 soon became indispensable.

The protocol of SR101 labeling was cheap and easily established in a laboratory, and could be used *in vivo* as well as in slice preparations from rostral brain region (Kafitz et al., 2008; see Figure 1A as an example). SR101 could be used to counterstain astrocytes when analyzing other cell types (Nimmerjahn et al., 2005) or for identification of astrocytes when analyzing electrophysiological properties of cells (Du et al., 2016) or together with calcium imaging of astrocytes (Pirttimaki and Parri, 2012). Furthermore, it initially appeared not to alter the physiological properties of brain cells.

First problems with uncritical usage of SR101 were revealed in hypoxic conditions when neuronal hemichannels are opened and SR101 can enter neurons (Thompson et al., 2006). Moreover, SR101 does not label astrocytes in brainstem slices as strong and specific as in the hippocampus or cortex (Schnell et al., 2015). This lighter staining intensity, together with some dye entering neurons makes interpretation of SR101 labeling unreliable in these brain regions (Schnell et al., 2012, 2015).

Additionally, it became evident that it was overlooked that SR101 can diffuse via gap junctions from astrocytes to oligodendrocytes, thereby impairing a reliable identification of astrocytes (Wasseff and Scherer, 2011; Hill and Grutzendler, 2014; Hagos and Hülsmann, 2016). Since gap junctions connect oligodendrocytes and astrocytes in many brain regions (Orthmann-Murphy et al., 2008; Griemsmann et al., 2015), it cannot be assumed that all SR101-labeled cells are astrocytes. In our hands, approximately 45% of SR101-positive cells did not express the fluorescent protein in the hippocampus of TgN (hGFAP-EGFP) mice (Schnell et al., 2012). Indeed, over 30% of SR101-labeled cortical cell were oligodendrocytes and, moreover, all mature oligodendrocytes in PLPcreER: mT/mG transgenic mice were reported to be SR101-labeled *in vivo* (Hill and Grutzendler, 2014).

## What Is the Uptake Mechanism of SR101-Labeling?

The accumulation of SR101 in astrocytes to a higher concentration compared to the extracellular solution indicated an active transport of SR101 into astrocytes in contrast to diffusion-based mechanism via gap junctions or hemichannels (Schnell et al., 2012). The pharmacological profile of SR101 pointed towards an organic anion transporting polypeptide (Schnell et al., 2012) and subsequent transcriptome analysis revealed region-specific differences in mRNA levels of the putative SR101 transporter, between brainstem and cortex/hippocampus, allowing the identification of the organic anion transporting polypeptide OATP1C1 (OATP14, OATPF, Slco1c1) as the responsible transporter for SR101 uptake into astrocytes (Schnell et al., 2015). The blockade of SR101-labeling by the unspecific gap junction blocker carbenoxolone (CBX) previously led to the conclusion that gap junctions are involved in the labeling of astrocytes with SR101. This is certainly true for the distribution of SR101 within the astroglial networks, but the complete blockade of SR101-labeling is probably caused by the blockade of the OATP1C1 by CBX (Nishimura et al., 2007; Schnell et al., 2012). Further evidence for this was the reduction of astroglial SR101-labeling by the organic anions MK-571 and Probenecid, which are known substrates of OATPs and would compete with SR101 for uptake via the OATP1C1 (Schnell et al., 2012). The ultimate proof comes from the blockade of SR101 labeling by the natural substrate of OATP1C1 levothyroxine (T4) and the absence of astrocytic SR101 labeling in OATP1C1-deficient mice (Schnell et al., 2015).

## Why Astrocytes Are Preferential Labeled?

**Figure 2:**
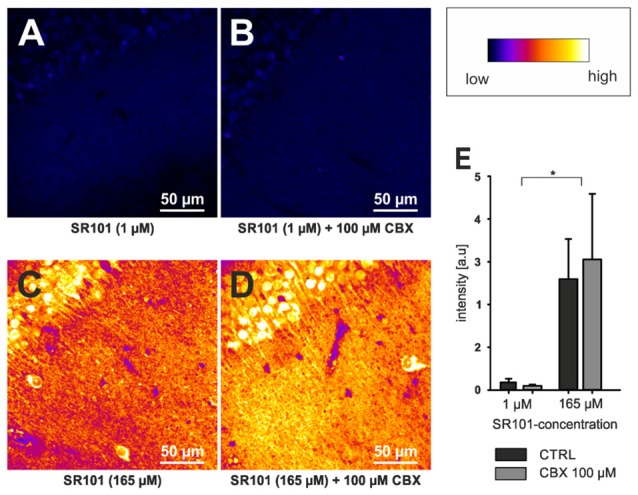
**Neuronal labeling in the CA1 region of the hippocampus by SR101 does not require OATP1C1. (A)** Labeling of slices from *Oatp1c1* knock out mice (Mayerl et al., 2012) using 1 μM SR101 for 20 min at 34°C followed 10 min of de-staining in aCSF (Schnell et al., 2012). **(B)** Same protocol except that 100 μM of carbenoxolone (CBX) was added during the staining procedure. Note that labeling of neurons was not reduced by CBX. In **(C,D)** the staining procedure was altered and 165 μM SR101 was applied at room temperature (Kantor et al., 2012). This protocol leads to a much brighter staining of neurons (see lookup table that was used for all four panels) but not to a staining of astrocyte-like cells. Again application of 100 μM CBX did not block the neuronal labeling. **(E)** Statistical analysis of the fluorescence intensity of neurons. Threshold based pixel analysis using ImageJ software. The asterisks indicated significance between 165 μM and 1 μM SR101 treatments. ANOVA with all pairwise multiple comparison procedures (Holm-Sidak method; *p* < 0.05; *n* = 3 mice) using SigmaPlot software.

Although OATP1C1 expression is a necessity for SR101 labeling of astrocytes (Schnell et al., 2015) and also oligodendrocytes (Hagos and Hülsmann, 2016; see also Figure 1B), its expression in astrocytes is not sufficient to explain the preferential labeling of astrocytes (and secondarily oligodendrocytes). At first, other cell types, such as endothelial cells express OATP1C1 (Lang et al., 2011; Ridder et al., 2011), and also neurons synthetize high levels of *Oatp1c1* mRNA (Cahoy et al., 2008). However, the functional expression levels of OATP1C1 protein in neurons are uncertain (Lang et al., 2011; Ridder et al., 2011). Interestingly, we found that neurons in the brainstem are loaded with SR101 during the staining procedure, leading to even higher fluorescence intensities in neurons as compared to neighboring astrocytes and the extracellular solution (Schnell et al., 2012). Yet, neurons are de-staining very quickly. Since this de-staining is blocked by MK-571, a blocker of ABC-transporters (Schnell et al., 2012), an additional differential expression of a yet unidentified transporter that mediates the extrusion of SR101 from neurons but not from astrocytes is required to explain these results. Since the time course of astrocyte labeling was slower as compared to neurons (Schnell et al., 2012), we also have to assume a different labeling process in neurons. Indeed, we found that SR101 labeling of superficial hippocampal neurons is still possible in the OATP1C1 knockout mouse or after application of levothyroxine (T4), a natural OATP1C1 substrate (Schnell et al., 2015). If this neuronal labeling was, as initially assumed, due to hypoxia-mediated hemichannels opening (Thompson et al., 2006), it should be prevented by application of a gap junction blocker. Although the intensity is slightly reduced, neurons in OATP1C1-deficent mice are still SR101-labeled after application of 100 μM CBX (Figures 2A,B,E). High concentration of SR101 (165 μM) applied at room temperature have been shown to preferentially label neurons in the hippocampus and *locus coeruleus* (Kantor et al., 2012). When testing this concentration in OATP1C1-deficient mice in the presence of CBX, we still observed neuronal labeling (Figures 2C,D), pointing towards an independent unknown SR101-uptake mechanism that is not present in astrocytes.

## Excitatory Side Effects of the Labeling

It seems obvious that a substance that interferes with a multi-specific transporter in the brain will cause side effects if applied at high concentrations. Indeed, recent reports of increased neuronal excitability challenged the usefulness of SR101 for functional analysis (Kang et al., 2010). Even at a concentration as low as 1 μM, SR101 increased the excitability in slices, including induction of LTP that outlasted the application time (Kang et al., 2010). Others reported similar effects only at higher SR101 concentration (Fink et al., 2012). *In vivo*, epileptic activity could be induced by intra-hippocampal injection of small volumes of 10 μM SR101 (Kang et al., 2010) or topical application of 100 μM (Rasmussen et al., 2016). The mechanism of SR101-induced hyper-excitability remains to be determined, though SR101-induced LTP was due to amplification of NMDA-receptor mediated currents (Kang et al., 2010).

The fact that SR101 uses OATP1C1, a thyroid hormone transporter (Sugiyama et al., 2003; Friesema et al., 2005), for entering the astrocytes, does not offer a plausible explanation for the changes of excitability alone. An increase of the extracellular levothyroxine (T4) or triiodothyronine (T3) levels induced by the competition of SR101 with their uptake transporter is expected to reduce neural excitability rather than causing hyper-excitability (Losi et al., 2008; Cao et al., 2011).

**Figure 3:**
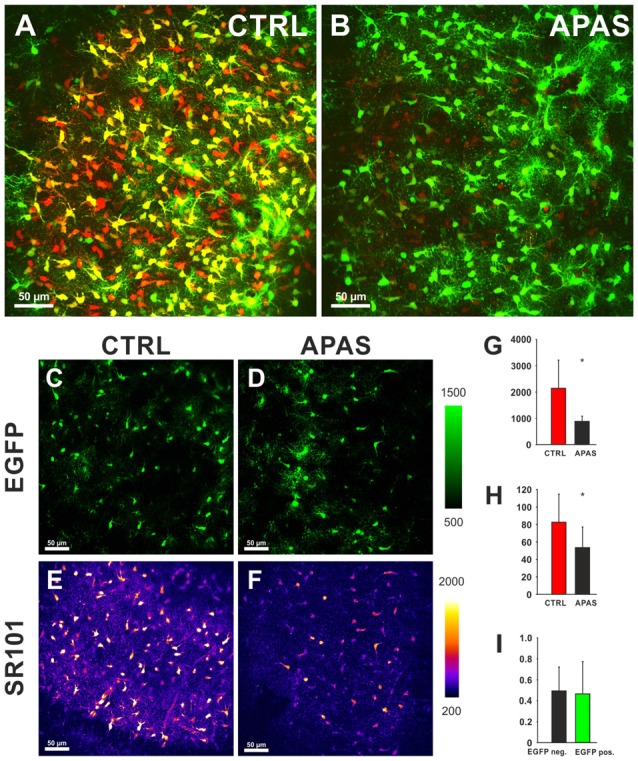
**Effects of neurosteroids on SR101 uptake in the hippocampus. (A)** Astroglial EGFP-fluorescence (green) and SR101-fluorescence (red) in the hippocampus after a staining in control conditions (1 μM SR101 for 20 min at 34°C). **(B)** Reduced SR101-labeling of hippocampal astrocytes when the neurosteroid allopregnanolone sulfate (APAS, 100 μM) was included in the staining solution. Both figures show maximum intensity projection 51 stacks (2 μm distance). **(C–F)** Images from single layer for EGFP **(C,D)** and SR101 **(E,F)**. Note the reduction of the intensity of SR101 after application of APAS **(F)**. **(G–I)** Statistical analysis: **(G)** The reduction of the SR101-intensity by 100 μM APAS is significant (Mann-Whitney Rank Sum Test; *p* = 0.032; *n* = 5 slices; 3 mice). **(H)** Additionally the number of SR101 positive cells than can be identified after APAS is reduced (*t*-Test; *p* = 0.040). **(I)** The APAS-induced reduction of SR101 intensity in EGFP-positive cells and EGFP-negative SR101 labeled cells is not different (Mann-Whitney Rank Sum Test; *p* = 0.548). Asterisks in **(G,H)** indicate significance (*p* < 0.05).

On the other hand, sulfated steroids (DHEAS, Estron-3-sulfate (Schnell et al., 2012), and allopregnanolone sulfate (3α, 5α-tetrahydroprogesterone sulfate; 3α, 5α-THP sulfate) blocked the uptake of SR101 into hippocampal and cortical astrocytes (Figure 3). We postulate that the competition of SR101 with the natural uptake of sulfated neurosteroids can change neuronal activity by enhancement of NMDA-receptors and inhibition of GABA_A_-receptors (Gibbs and Farb, 2000). Since neurosteroids levels do vary in the brain depending on different factors like stress and estrus (Paul and Purdy, 1992) it is difficult to predict if changes in excitability are induced by SR101 at a certain experimental condition. On the other hand, it is tempting to speculate that such variation in neurosteroids levels can explain that side effects have been seen by some groups but not by others.

## Conclusion

Given the limitations of the SR101 as mentioned above and summarized in Table 1, it is necessary to give a statement of caution. The use of SR101, without being aware of the caveats regarding cell type specificity and possible side effects, might affect the validity of research. Therefore, researchers should be encouraged to employ additional measures like electrophysiological whole-cell recordings of SR101-labeled cells and *post hoc* immunohistochemistry to confirm the specificity of SR101 staining in their experimental setting. To minimize excitatory side effects, the concentration of SR101 has to be kept as low as possible or the labeling procedure could be performed after the actual experiment.

**Table 1:** **The table summarizes labeling procedures that have been found to be not specific for astrocytes**.

Protocol	Cell types labeled	Reference	Excitatory side effects reported
Slice; 10–140 min incubation at 34°C, 0.5–1 μM SR101	Astrocytes and oligodendrocytes	Wasseff and Scherer (2011), Hagos and Hülsmann (2016)	Long-term negative shift of AP threshold, LTP; ≥1 μM SR101 for 10 min; (Kang et al., 2010); LTP ≥25 μM SR101 for 10 min SR101 (Fink et al., 2012)
Slice; incubation at RT (20–23°C), 165 μM SR101	Neurons	Kantor et al. (2012), this article	–
Slice, incubation and OGD at RT, 100 μM SR101	Neurons	Thompson et al. (2006)	–
*In vivo*; 100 μL intravenously 5 mM SR101in PBS	Astrocyte and oligodendrocytes	Hill and Grutzendler (2014)	–
*In vivo*; topically to cortical surface for 5–10 min, 50 μM SR101	Astrocytes and oligodendrocytes	Hill and Grutzendler (2014)	Seizure-like activity, ≥100 μM SR101 for 10 min (Rasmussen et al., 2016)

*RT, room temperature; OGD, oxygen glucose deprivation; PBS, Phosphate buffered saline; LTP, Long term potentiation*.

## Ethics Statement

In accordance with the German Protection of Animals Act (Tierschutzgesetz; TierSchG §4 Abs. 3) all procedures were approved by the Animal Welfare Office of University Medical Center (file number T12/11).

## Author Contributions

SH and CS designed experiments; SH, CS and LH conducted experiments; SH, CS and HH wrote the manuscript.

## Funding

The study was supported by the Cluster of Excellence and Deutsche Forschungsgemeinschaft (DFG) Research Center Nanoscale Microscopy and Molecular Physiology of the Brain (CNMPB). We acknowledge support by the Open Access Publication Funds of the Göttingen University.

## Conflict of Interest Statement

The authors declare that the research was conducted in the absence of any commercial or financial relationships that could be construed as a potential conflict of interest.
